# Protocol of a phase II study to evaluate the efficacy and safety of deep-inspiration breath-hold daily online adaptive radiotherapy for centrally located lung tumours (PUDDING study)

**DOI:** 10.1186/s13014-024-02427-4

**Published:** 2024-03-08

**Authors:** Noriko Kishi, Masahiro Yoneyama, Hiroyuki Inoo, Minoru Inoue, Hiraku Iramina, Akiyoshi Nakakura, Tomohiro Ono, Hideaki Hirashima, Takanori Adachi, Norimasa Matsushita, Makoto Sasaki, Takahiro Fujimoto, Mitsuhiro Nakamura, Yukinori Matsuo, Takashi Mizowaki

**Affiliations:** 1 Department of Radiation Oncology and Image-Applied Therapy, Graduate School of Medicine, Kyoto University, 54 Shogoin-Kawahara-Cho, Sakyo-Ku, Kyoto, 606-8507 Japan; 2 Department of Biomedical Statistics and Bioinformatics, Graduate School of Medicine, Kyoto University, 54 Shogoin-Kawahara-Cho, Sakyo-Ku, Kyoto, 606-8507 Japan; 3 Clinical Radiology Service, Kyoto University Hospital, Kyoto, Japan; 4 Department of Information Technology and Medical Engineering, Division of Medical Physics, Graduate School of Medicine, Human Health Sciences, Kyoto University, Kyoto, Japan; 5 Department of Radiation Oncology, Faculty of Medicine, Kindai University, 377-2, Onohigashi, Osakasayama-Shi, Osaka, 589-8511 Japan

**Keywords:** Primary lung tumour, Metastatic lung tumour, Deep-inspiration breath-hold, Adaptive radiotherapy

## Abstract

**Background:**

Centrally located lung tumours present a challenge because of their tendency to exhibit symptoms such as airway obstruction, atelectasis, and bleeding. Surgical resection of these tumours often requires sacrificing the lungs, making definitive radiotherapy the preferred alternative to avoid pneumonectomy. However, the proximity of these tumours to mediastinal organs at risk increases the potential for severe adverse events. To mitigate this risk, we propose a dual-method approach: deep inspiration breath-hold (DIBH) radiotherapy combined with adaptive radiotherapy. The aim of this single-centre, single-arm phase II study is to investigate the efficacy and safety of DIBH daily online adaptive radiotherapy.

**Methods:**

Patients diagnosed with centrally located lung tumours according to the International Association for the Study of Lung Cancer recommendations, are enrolled and subjected to DIBH daily online adaptive radiotherapy. The primary endpoint is the one-year cumulative incidence of grade 3 or more severe adverse events, as classified by the Common Terminology Criteria for Adverse Events (CTCAE v5.0).

**Discussion:**

Delivering definitive radiotherapy for centrally located lung tumours presents a dilemma between ensuring optimal dose coverage for the planning target volume and the associated increased risk of adverse events. DIBH provides measurable dosimetric benefits by increasing the normal lung volume and distancing the tumour from critical mediastinal organs at risk, leading to reduced toxicity. DIBH adaptive radiotherapy has been proposed as an adjunct treatment option for abdominal and pelvic cancers. If the application of DIBH adaptive radiotherapy to centrally located lung tumours proves successful, this approach could shape future phase III trials and offer novel perspectives in lung tumour radiotherapy.

*Trial registration*. Registered at the Japan Registry of Clinical Trials (jRCT; https://jrct.niph.go.jp/); registration number: jRCT1052230085 (https://jrct.niph.go.jp/en-latest-detail/jRCT1052230085).

## Backgrounds

Treatment of centrally located lung tumours positioned around the trachea/bronchus, whether primary or metastatic, is challenging. Such tumours are more susceptible than peripheral lung tumours to eliciting symptoms such as airway obstruction, atelectasis, and bleeding. Surgical resection, including pneumonectomy, bronchial sleeve resection, and plasty, is a common treatment strategy. Pneumonectomy carries a 2–3 times higher risk of postoperative complications compared to standard lobectomy, placing a significant strain on the circulatory and respiratory systems. Sleeve resection, in contrast, requires advanced techniques as it necessitates cutting the vessels and bronchi, excising the lobes containing the lesion, and subsequently re-anastomosing. Consequently, definitive radiotherapy is the preferred option for patients who are unsuitable for surgical resection or those with high-risk surgical comorbidities [1].

As a definitive radiotherapy technique, stereotactic body radiotherapy (SBRT), which precisely delivers a high irradiation dose to extracranial targets in just one or a few treatment fractions, has been applied to centrally located lung tumours [2]. SBRT improves local control compared with conventional three-dimensional conformal radiotherapy [3]. However, SBRT for centrally located lung tumours has a higher risk of severe adverse events than for peripherally located tumours (34% vs. 10%) because the tumour is located adjacent to the mediastinum, including radiosensitive organs at risk (OARs) such as the oesophagus, trachea, and bronchus [4, 5]. To reduce these severe adverse events (AEs), it is imperative to decrease the irradiation doses to OARs. To achieve this, we conduct a phase II study employing two combined approaches.

The first approach is deep inspiration breath-hold (DIBH) radiotherapy, which has been utilised to reduce respiratory motion, thereby reducing the irradiated volume of the OARs. In peripheral lung tumours, especially those in the lower lobe, DIBH radiotherapy plays a key role in reducing respiratory motion; however, its role has been negligible in centrally located tumours. However, a recent study indicates that DIBH not only minimises respiratory motion but also extends the gap between the tumour and adjacent normal OARs by 3.8 mm, attributable to the expansion of the normal lung [6]. This method can reduce the maximum dose to normal OARs by decreasing the irradiated volume and creating a distinct separation between the tumour and adjacent OARs.

The second approach involves adaptive radiotherapy, which is characterised by changing the radiation treatment plan delivered to a patient during the course of radiotherapy to account for either temporal changes in anatomy (e.g. tumour size, internal motion, variations in respiratory patterns, and weight loss) or changes in tumour biology/function [7]. When applying adaptive radiotherapy to centrally located lung tumours, interfractional variations at end inhalation is adjusted by daily adaptation, and the margin size can be decreased [8, 9]. This would lead to improved local control by delivering a high dose to the tumour while simultaneously decreasing the irradiated dose to adjacent normal OARs. In line with this, a phase II trial utilising daily online adaptive magnetic resonance image-guided SBRT in ultracentrally located lung tumours, named the STAR-LUNG STUDY (NCT05354596), is currently in progress in Denmark.

Currently, no clinical trials have employed both DIBH and daily online adaptive radiotherapy simultaneously for centrally located lung tumours. In our study, by integrating these techniques, we aim to amplify the physical separation between the lung tumour and normal OARs and to refine and reduce the irradiated volume and dose distribution.

## Methods and design

### Objectives

In radiotherapy, increasing the dose per fraction and completing the scheduled treatment in a short duration can elevate local control rates. However, when treating centrally located lung tumours, there are inherent risks such as bleeding. This necessitates a reduction in dose per fraction and an increase in fractionation. DIBH in combination with daily online adaptive radiotherapy addresses these challenges. Even if the positions of the internal organs change during each treatment session, adjusting the irradiation field becomes feasible. This method guarantees that the tumour receives the intended dose while actively sparing the surrounding normal OARs. In the future, if we can administer an ablative dose per fraction in a shorter treatment period, we could improve the local control of centrally located lung tumours without increasing the risk of AEs.

This study aims to evaluate the efficacy and safety of DIBH radiotherapy in combination with online adaptive radiotherapy for centrally located lung tumours.

### Study design and follow-ups

This is a single-arm, prospective phase II trial conducted at a single institution. Eligible patients meeting the inclusion criteria are enrolled.

**Fig. 1:**
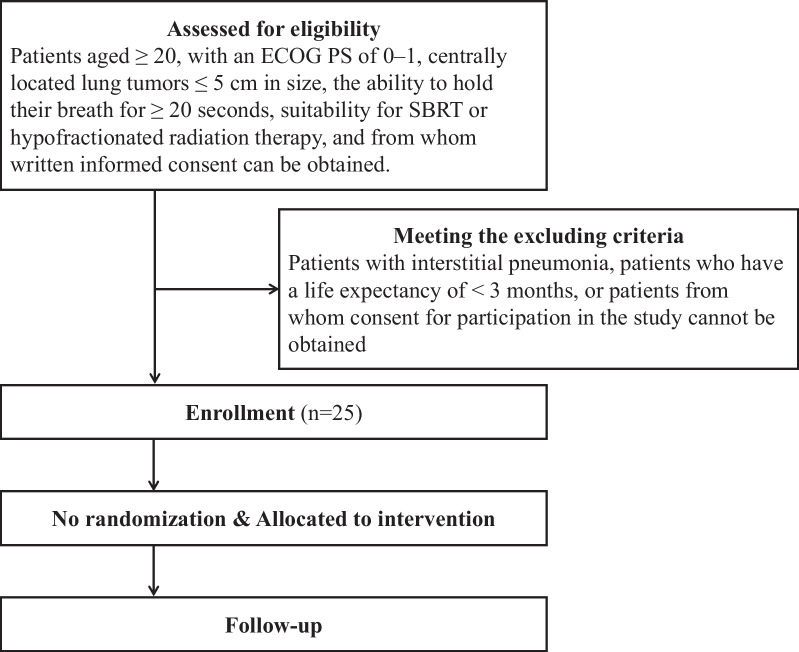
The protocol schema of this study

Before the initiation of scheduled treatment, we assess the patient’s general condition using the Geriatric 8 screening tool [G8], Eastern Cooperative Oncology Group Peformance Status (ECOG-PS), height, and body weight. Imaging examinations are performed, including chest X-ray, chest CT, FDG-PET, and brain MRI with contrast if possible. We conduct physiological tests, such as pulmonary function assessments, as well as blood tests to check white blood cell count, haemoglobin, platelets, creatinine, total bilirubin, albumin, C-reactive protein, electrolytes (sodium, potassium, and chloride), and tumour markers according to the histological type of the primary lesion. Quality of life is assessed using EORTC-QLQ-C30 and EORTC-QLQ-LC1 3 questionnaires, while toxicities ≥ grade 2 are assessed using the Common Terminology Criteria for Adverse Events version 5.0 (CTCAE v5.0) guidelines. Finally, we conduct therapeutic efficacy evaluations at baseline. During treatment, if grade 2 or higher severe AEs (CTCAE v5.0) are observed, the worst grade and date of diagnosis are recorded. Patients have follow-up visits every 3 months during the first-year post-treatment. These visits include assessments of their general condition, imaging examinations, blood tests, quality of life evaluations, evaluation of grade 2 toxicities (CTCAE v5.0), and therapeutic efficacy assessments. The study calendar is detailed in Table 1, and the protocol schema is illustrated in Figure 1.

**Table 1:** Study calendar of this study

	Before registration	Before treatment	During treatment	After treatment (months)				> 1 year
				3	6	9	12	
Patient background	O							
General condition	O	O		O	O	O	O	As needed
Imaging examination	O			O	O	O	O	As needed
Physiological test	O							As needed
Blood test	O			O	O	O	O	As needed
Quality of life		O		O	O	O	O	
Toxicities ≥ grade 2 (CTCAE v5.0)			O	O	O	O	O	As needed
Therapeutic efficacy evaluation				O	O	O	O	As needed

*CTCAE* Common Terminology Criteria for Adverse Events

### Ethics

The study was conducted in accordance with the Declaration of Helsinki (revised in 2013). This study was approved by the institutional review board (IRB) of Kyoto University (IRB number: Y0156). All participants or their authorized representatives will receive detailed information about the nature of the study and must provide written informed consent before being enrolled. Patients enrolled in this study will engage in discussions with healthcare providers to understand the risks, benefits, and intervention details. This study was registered in the Japan Registry of Clinical Trials (Trial registration number: jRCT1052230085).

### Inclusion criteria

Inclusion criteria for this study are as follows:

1. Aged 20 years or older.
2. ECOG-PS of 0 or 1
3. Centrally located lung tumours with a maximum diameter of 5 cm or less, defined according to the IASLC recommendations as tumours located within 2 cm of the mediastinum (including bronchi, oesophagus, heart, brachial plexus, major blood vessels, spinal cord, phrenic nerve, and recurrent laryngeal nerve) [10].
4. Patients who can hold their breath for more than 20 s.
5. Patients who are considered suitable for stereotactic body radiation therapy meet the predefined dose constraints for OARs, as determined by board-certified radiation oncologists.
6. Patients who provide written informed consent.

### Exclusion criteria

Exclusion criteria for this study are as follows:

1. Patients with interstitial pneumonia
2. Life expectancy of less than 3 months according to estimations by board-certified radiation oncologists
3. Patients who are not capable of giving consent.

### Interventions

#### Treatment planning

The patient is immobilised in the supine position with both arms raised using BodyFIX (Elekta, Stockholm, Sweden). DIBH CT images is obtained using the SDX system (DYN’R, Toulouse, France), which is a respiratory monitoring and measurement system. These images serve as a basis for treatment planning and will be obtained using a 64-slice CT scanner (SOMATOM Definition AS; Siemens Healthineers, Erlangen, Germany). As an alternative treatment plan, 4D-CT scans is performed under free breathing without the SDX system and with the Real-time Position Management system (RPM; Varian Medical Systems, Palo Alto, CA, USA). If deemed clinically necessary, contrast agents is administered. Eclipse version 15.6 (Varian Medical Systems) and later versions are used for treatment planning.

The gross tumour volume (GTV) is delineated based on the DIBH CT and 4D-CT images. The clinical target volume (CTV) is identical to the GTV. A three-dimensional margin of approximately 3–6 mm is added to the CTV to define the planning target volume (PTV), depending on the setup accuracy and internal margin considering tumor’s location.

#### Dose prescription and dose constraints for OARs

In the Japanese phase I trial of central lung cancer, JROSG10-1, the maximum tolerated dose is established at 60 Gy in 8 fractions, prescribed at the isocentre [11]. In the phase I trial, D_95%_ of the PTV is 52.3 Gy (range, 43.2–55.9 Gy), and the maximum dose reaches 103.7% (range, 100–107%). Our study adopts a D_95%_ volume prescription strategy, where D_X%_ refers to delivering the prescribed dose to X% of the PTV volume. Ongoing phase II trials for centrally located lung tumors, STRICT-LUNG STUDY and STAR-LUNG STUDY (NCT05354596), utilize PTV D_95%_ ≥ 53.2 Gy. Accordingly, 54 Gy in 8 fractions is selected for PTV D_95%_ prescription, aligning closely with the near-equivalent dose in the maximum tolerated dose identified in JROSG10-1 and analogous to practices in other phase II trials.

Using the RTOG 1106 atlas, we contour the OARs [12]. For serial OARs, a planning organ at risk volume is generated, which is determined with a margin of 3 mm added to the OAR, including the spinal cord, brachial plexus, oesophagus, heart, aorta, stomach, etc. Dose constraints are set based on JROSG10-1 [11], as shown (Table 2).

**Table 2:** Dose constraints for OARs

OARs	Indices	Dose (Gy)	Toxicities for endpoints
Spinal cord	Max (D_0.03cc_)	33.5	Myelitis
Ipsilateral brachial plexus	Max (D_0.03cc_)	40	Neuropathy
Skin*	Max (D_0.03cc_)	40	Skin ulcer
Oesophagus	< 5 cc	40	Stenosis/fistula
Heart/Pericardium	< 15 cc	40	Pericarditis
Aorta	< 10 cc	58	Aneurysm
Pulmonary artery	< 1 cc	54.5	Aneurysm
	< 10 cc	47.5	
Superior vena cava, pulmonary vein	< 1 cc	48	Stenosis/Fistula
Trachea/Bronchus	< 10 cc	54.5	Stenosis/Fistula
Lungs (Bilateral lungs-PTV)*	V_20Gy_ < 20%	–	Pneumonia
Stomach and intestines^†^	Max (D_0.03cc_)	45	Bleeding/Ulcer/Fistula
	< 10 cc	40	

*OAR* organs at risk; *PTV* planning target volume; *V*_*20Gy*_ percentage of OAR volume receiving 20 Gy or more

*Indices and doses are calculated based on the planning organ at risk for each organ, except for the lungs and skin

^†^Dose constraints for stomach and intestines are determined based on SUNSET trial [15]

#### Irradiation techniques

Ethos (Varian Medical Systems), a linear accelerator equipped with 6 MV-FFF X-rays, is utilised. Coplanar multiple-photon beams or arcs with intensity modulation is used to create the treatment plan. The DIBH technique using the SDX system is employed for cone-beam CT (CBCT) imaging for positioning and treatment planning, as well as for each treatment session.

In the online adaptive radiotherapy approach, automatic contouring of PTV and OARs typically does not require manual modification. A new treatment plan is generated based on the daily first DIBH CBCT, referred to as an adapted plan. This adapted plan is compared with the original treatment plan, a scheduled plan, created using the treatment planning DIBH CT. The scheduled plan is utilized for treatment delivery when the adapted plan does not meet the dose prescription for PTV or the predefined dose constraints for the OARs, and it can achieve a lower irradiated dose to OARs compared with the adapted plan.

After determining which to use the adapted plan or the scheduled plan, the daily second DIBH CBCT is performed immediately before treatment delivery. This ensures that any patient positional errors or internal organ displacements that occurred during the preparation of the new treatment plan fall within acceptable margins.

#### Endpoints

The primary endpoint is the occurrence of grade 3 or higher severe adverse events (CTCAE v5.0) within one-year of treatment. The secondary endpoints are overall survival rate, disease-free survival rate, local recurrence-free survival rate, quality of life, grade 2 or more severe AEs, 1-year cumulative incidence of grade 2 or more severe radiation pneumonitis, and the number of patients and fractions requiring adapted plans. Data on dose-volume indices, structures of target volumes and OARs, set-up error, treatment time, quality assurance of planned online adaptive radiotherapy treatment, medical images, and ventilation volume are also collected. If multiple AEs occurr in the same patient, they are classified according to the highest severity.

### Statistical analysis and sample size estimation

The overall survival rate, disease-free survival rate, local recurrence-free survival rate, and 1-year cumulative incidence are calculated using the Kaplan-Meier method. The incidence of grade 3 or more severe AEs in DIBH online adaptive radiotherapy is assumed to be similar to that of peripheral lung tumours, 10% [5]. We set 33% as a threshold for the Nordic HILUS trial [4]. At a one-sided significance level of 5% and power of 80%, the sample size is estimated to be 20 patients. The estimated number of patients with centrally located lung tumours treated with SBRT at our institute, who meet the inclusion criteria on age, ECOG-PS, tumor location, and size, is approximately 20 per year. We aim to enroll 25 patients over a three-year period, factoring in a 40% consent rate and accounting for potential dropouts.

## Discussion

This PUDDING study is conducted to evaluate the efficacy and safety of DIBH radiotherapy in combination with daily online adaptive radiotherapy for centrally located lung tumours. If the results demonstrate that daily DIBH online adaptive radiotherapy is safe and feasible, further investigation is warranted in a phase III trial to assess its efficacy. Although several phase I–II trials for centrally located lung tumours have previously demonstrated the efficacy and toxicity of SBRT [4, 11, 13–15], they did not focus on DIBH or adaptive radiotherapy. Therefore, optimal strategies for the treatment of centrally located lung cancer have not yet been established, and there is room for improvement.

However, the optimal fractionation of centrally located lung tumours remains to be elucidated. Previous trials employed SBRT scheduled in 5–8 fractions; the 2-year local control rates were 88–89% for 5 fractions and 83% for 8 fractions [4, 13]. A dose-control relationship has been observed between the prescribed dose and local control [16, 17]. However, increasing the dose to the PTV results in a trade-off with an increased risk of AEs, as the PTV is located adjacent to the OARs in centrally located lung tumours. Centrally located lung tumours are commonly defined as tumours located 2 cm from the proximal bronchial tree or tumours adjacent to the mediastinal or pericardial pleura, with grade 3 or more severe AEs observed in 0–7.2% of cases [11, 13]. A phase II Nordic HILUS trial, which adopted the definition that tumours located within 1 cm of the proximal bronchial tree are centrally located lung tumours, reported that 34% of patients developed grade 3 or more severe AEs [2]. Of these, 15% experienced treatment-related death. Given these safety data, we adopted the IASLC definition, which classifies tumours located within 2 cm of the mediastinum as central tumours. This definition facilitates appropriate patient selection in the current study (8 fractions), aiming to reduce the risk of AEs while maintaining the PTV dose.

DIBH radiotherapy offers dosimetric advantages by increasing the volume of the normal lung and expanding the distance between the tumour and the OARs. These dosimetric benefits correlate clinically with a significant reduction in acute pulmonary toxicity as well as late pulmonary, cardiac, and oesophageal toxicities [18]. Appels et al. reported that DIBH with continuous positive airway pressure (CPAP) ventilation not only minimised the respiratory motion of the lung tumour but also extended the distance between the tumour and mediastinal OARs by 3.8 mm, which is attributable to the expansion of the normal lung [6]. Their findings showed a 1.5% rate of grade 3 AEs and a 2-year local control rate of 90% in patients with central lesions. They proposed that CPAP-assisted DIBH lung SBRT was promising when free breathing or standard DIBH radiotherapy alone might be unsafe or ineffective. As CPAP is not suitable for cases of respiratory failure or pulmonary cysts, a simple DIBH technique with adjustment via daily online adaptive radiotherapy can be employed for those who are not amenable to CPAP-DIBH. Considering breath-hold adaptive radiotherapy is promising in terms of sparing OARs and improving local control for abdominal and pelvic cancers [19–21], it would become a feasible option to expand the range of treatment alternatives for lung tumours.

In conclusion, this PUDDING study is a pivotal effort to evaluate the combined efficacy of DIBH and daily online adaptive radiotherapy for centrally located lung tumours. The concurrent application of DIBH and daily online adaptive radiotherapy has been unprecedented in previous trials, thus positioning this study as an innovative exploration in this field. If the results are promising, this approach could serve as a foundation for further investigations in a phase III trial. The significance of this study lies in its potential to provide new insights into lung tumour treatment strategies.

## Data Availability

Full trial protocol was made available by contacting Noriko Kishi (kishin@kuhp.kyoto-u.ac.jp).

## Abbreviations

AEs: Adverse Events
CPAP: Continuous positive airway pressure
CTCAE: Common Terminology Criteria for Adverse Events
CTV: Clinical target volume
DIBH: Deep-inspiration breath-hold
ECOG-PS: Eastern Cooperative Oncology Group Performance Status
GTV: Gross tumour volume
IRB: Institutional review board
OAR: Organs at risk
PTV: Planning target volume
RPM: Real-time Position Management
TV: Target volume
